# Towards a Point-of-Care Test of CD4^+^ T Lymphocyte Concentrations for Immune Status Monitoring with Magnetic Flow Cytometry

**DOI:** 10.3390/mi15040520

**Published:** 2024-04-13

**Authors:** Moritz Leuthner, Mathias Reisbeck, Michael Helou, Oliver Hayden

**Affiliations:** 1 Heinz-Nixdorf-Chair of Biomedical Electronics, School of Computation, Information and Technology & Munich Institute of Biomedical Engineering, Technical University of Munich, TranslaTUM, Einsteinstraße 25, 81675 Munich, Germany; 2 EarlyBio GmbH, Bottroper Weg 2, 13507 Berlin, Germany

**Keywords:** magnetic flow cytometry, point-of-care testing (POCT), immune status, whole blood, lab-on-a-chip (LOC), human immunodeficiency virus (HIV), flow cytometry

## Abstract

For the treatment of human immunodeficiency virus (HIV)-infected patients, the regular assessment of the immune status is indispensable. The quantification of CD4^+^ T lymphocytes in blood by gold standard optical flow cytometry is not point-of-care testing (POCT) compatible. This incompatibility is due to unavoidable pre-analytics, expensive and bulky optics with limited portability, and complex workflow integration. Here, we propose a non-optical, magnetic flow cytometry (MFC) workflow that offers effortless integration opportunities, including minimal user interaction, integrated sample preparation and up-concentration, and miniaturization. Furthermore, we demonstrate immunomagnetic CD4^+^ T lymphocyte labeling in whole blood with subsequent quantification using sheath-less MFC. Showing linearity over two log scales and being largely unimpaired by hematocrit, evidence is provided for POCT capabilities of HIV patients.

## 1. Introduction

The initial idea of lab-on-a-chip (LOC) systems is the combination of microchip technology for electronics with chemistry [1,2]. Here, we expanded this approach towards a medical workflow for quantitative blood cell analysis at the point-of-care with a miniaturized flow cytometer comprising a microelectronic chip with an attached microfluidic channel.

CD4^+^ T lymphocytes are an established biomarker for assessing a person’s immune competence. For patients with a preceding human immunodeficiency virus (HIV) infection, the monitoring of their CD4^+^ T lymphocyte concentration is vital for treatment planning and disease monitoring [3]. The typical CD4^+^ T lymphocyte concentration in peripheral blood ranges from up to ~1400 µL^−1^ in healthy persons to less than 100 µL^−1^ in HIV-infected patients [4,5]. Upon initial patient assessment, the CD4^+^ T lymphocyte concentration is the most essential laboratory indicator for the patient’s immune system function and is a strong predictor of the disease’s progression and survival [6,7]. During antiretroviral therapy (ART), the therapy’s success on the immune system should be evaluated typically every 3 to 6 months, especially to verify the CD4^+^ T lymphocyte concentration increase in a minimum of 50 µL^−1^ in the first year until a steady CD4^+^ T lymphocyte concentration has been established [4,8].

Today’s gold standard for determining the patient’s CD4^+^ T lymphocyte concentration is optical flow cytometry (OFC) [9]. However, due to tedious sample preparation and logistics by well-trained users, high instrument complexity, and limited access at centralized laboratories, OFC is not established for point-of-care testing (POCT) [10]. This is mainly due to the OFC’s major bottlenecks of the cellular background of blood, e.g., erythrocytes, impeding the optical readout, thus demanding cumbersome and user-dependent pre-analytics, e.g., cell lysis, high instrument costs determined by the optical components, and limited portability [11,12,13,14,15]. Magnetic flow cytometers (MFC), typically micrometer-scaled, solid-state devices integrated into microfluidic systems, exhibit advantages compared to OFCs, such as the fact that non-optical probing of samples is insensitive to biological background. Therefore, fluorophore labels are substituted by magnetic nanoparticles (MNP), lasers and optical detectors are replaced with Hall effect, giant or tunnel magnetoresistive (GMR, TMR) sensors embedded into the microfluidic channel, and sample preparation and enrichment can be provided directly on-chip [16,17,18,19,20,21,22,23]. These advantages allow for miniaturization, highly integrated workflows, inexpensive production, robustness, and user-friendly operation of MFCs with POCT opportunities.

Since immune status assessments by determining the CD4^+^ T lymphocyte concentration in the blood are lifelong recurring for HIV patients, its POCT accessibility, especially in rural areas and developing countries with limited access to central laboratories, represents a medical need [24].

This article presents an MFC approach towards an immune status POCT solution. Our concept includes the immunomagnetic labeling and MFC measurement of CD4^+^ T lymphocytes in their natural whole blood (WB) environment. Therefore, we investigated the cellular background influence on the linearity of concentration measurements. Finally, we demonstrate POCT applicability by measuring clinically relevant CD4^+^ T lymphocyte concentrations in WB.

## 2. POCT Workflow Concept for Immune Status Assessment with MFC

**Figure 1:**
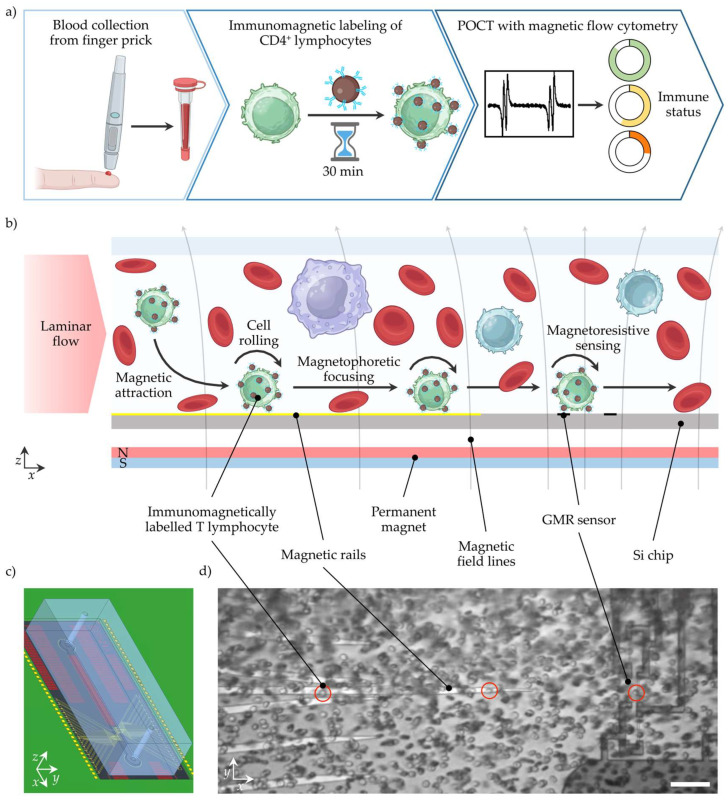
Workflow for POCT of CD4^+^ lymphocyte concentrations with MFC to determine the patient’s immune status. (**a**) Blood is collected from a finger prick and collected for dosing and further handling in a capillary. In the following, the WB sample is mixed with antibody-coated MNPs. After a short incubation time, CD4^+^ lymphocytes are immunomagnetically labelled. Next, the incubated sample is transported over a magnetoresistive sensor that detects the magnetized CD4^+^ lymphocytes. After signal analysis, the immune status can be inferred from the CD4^+^ lymphocyte concentration. (**b**) The WB sample is transported through a channel in a laminar flow. A permanent magnet creates a magnetic field density gradient that attracts the magnetized CD4^+^ lymphocytes towards the sensor surface. Magnetic rails in a chevron-like configuration facilitate magnetophoretic focusing of the magnetized CD4^+^ lymphocytes on the GMR sensors. When the magnetized cell passes over the GMR sensors in a Wheatstone half-bridge configuration, a four-peak signal is generated. (**c**) Rendered assembly of PCB, Si chip, and PDMS channel. (**d**) Image from a Si chip with immunomagnetically labelled CD4^+^ lymphocytes (circled in red) in 1:30 diluted WB passing through the channel. The scale bar represents 50 µm.

For POCT, the complete workflow from sample collection to data evaluation and interpretation is key. Typical settings for POCT include uncontrolled test environments, untrained users, limited medical assistance, lack of time, and minimal costs per test. Clinically relevant decisions should be inferred from the test result, which could significantly impact the treatment outcome. Therefore, minimal user interaction, robust processes, and low sample consumption are essential. In Figure 1a, the MFC workflow to determine the patient’s immune status from CD4^+^ T lymphocyte concentration is shown. In the first step, blood is collected from a finger prick and dosed using a capillary with an anti-coagulant coating to stabilize the blood. In diabetes care, blood collection from a finger prick is an established and accepted technique, while the capillary provides a safe container to transfer the blood sample to the next step [25]. In capillary blood samples, the CD4^+^ T lymphocyte concentration is equivalent to venous blood samples [26]. However, the capillary could also be implemented in a single-use cartridge or the device’s sample uptake unit [23]. Next, the blood sample is mixed with anti-CD4 antibody-coated MNPs to label the CD4^+^ T lymphocytes immunomagnetically. After a short incubation time of <30 min, the sample might be diluted by tens of magnitudes with a buffer, and is subsequently ready for the non-optical MFC measurement. For convenience, the MNPs could be deposited in a cartridge in a lyophilized form and rehydrated by the sample, similarly to the buffer. With this, no additional user interaction, cell lysis, or manual pre-processing is needed. After automated signal analysis, the CD4^+^ T lymphocyte concentration and immune status rating are displayed.

The MFC measurement is outlined in more detail in Figure 1b. A laminar, sheath-less flow transports the sample pulsation-free through a microfluidic channel. A permanent magnet below the microstructured Si chip generates a static magnetic field density gradient that pulls the magnetized CD4^+^ T lymphocyte towards the Si chip surface. Upon cell contact with the surface, the CD4^+^ T lymphocyte moves with a rolling motion over the surface. Besides a CD4^+^ T lymphocyte enrichment in the *z*-direction, integrated ferromagnetic rails on the silicon (Si) chip precisely guide the CD4^+^ T lymphocytes towards the GMR sensors, resulting in a second enrichment step (*y*-direction). Finally, when the magnetized CD4^+^ T lymphocyte passes the GMR sensors, a distinct signal pattern is recorded for each cell. The cellular background and unbound magnetic nanoparticles freely floating in the matrix have a negligible magnetic moment. They are, therefore, neither attracted by the magnetic field density gradient, nor induce a signal of the GMR sensor.

## 3. Materials and Methods

### 3.1. Magnetic Flow Cytometer

The customized Si chips comprising the sensing elements were fabricated by Sensitec GmbH (Wetzlar, Germany). The GMR sensors were configured in a temperature-insensitive Wheatstone half-bridge whilst each element had the size of 2 × 30 µm^2^. Additionally, magnetic rails made from nickel-iron were fabricated in a chevron pattern on the Si surface to focus magnetized objects precisely on the GMR sensors. The Si chip was fitted in a matching rectangular cut in a printed circuit board (PCB) and fixed with a glass slide glued with double-sided adhesive tape on the PCB’s bottom (see Supplementary Figure S3). Wire bonding from the Si to the PCB bond pads established the electrical connection between the PCB and the sensor. In order to attract magnetized objects onto the Si chip surface, a neodymium-iron-boron (NdFeB) permanent magnet (32 × 27 × 6 mm^3^) was positioned underneath the sensor, generating a vertical magnetic field density B_ext_ between 120–150 mT in the sensor plane [18].

The sample transport over the GMR sensors was facilitated with a poly(dimethylsiloxane) (PDMS) channel of 700 µm width and 150 µm height. This channel was fabricated using standard soft photolithography processes using epoxy resin (SU-8 2050, Kayaku Advanced Materials Inc., Westborough, MA, USA), as described elsewhere [27]. Inlet and outlet ports were punched into the PDMS with a biopsy punch before assembly. Insertion of poly(tetrafluoroethylene) (PTFE) tubing (RCT-ZS-DKA-SW 0.3 mm inner diameter, RCT Reichelt Chemietechnik GmbH + Co., Heidelberg, Germany) with a connection to a glass syringe (1750 TLL, Hamilton Company Corp., Reno, NV, USA) completed the fluidic system. Pulsation-free laminar flow conditions were established with a syringe pump (Fusion 4000, Chemyx Inc., Stafford, TX, USA).

To maximize the GMR sensor sensitivity, the external magnetic field from the magnet needed to be aligned with the GMR sensors (Figure 1b)) [17]. Due to the sensor being susceptible to solely *x-y*-components, the in-plane component of the magnetic field density (*x-y*-direction) was minimized by moving the magnet in *x-y*-direction while recording hysteresis loops from an alternating magnetic field (±15 mT) that is parallel to the sensor plane generated by Helmholtz coils (see Supplementary Figure S2). Finally, GMR effects between 7.1–7.5% and sensitivities between 1.2–1.4% mT^−1^ were achieved.

A 10 kHz modulated signal with a peak-to-peak voltage between 0.15–0.4 V (MFLI 500 kHz Lock-in Amplifier, Zurich Instruments AG, Zurich, Switzerland) supplied the Wheatstone half-bridge. The recording of the differential signal from the Wheatstone half-bridge was filtered with a 3rd-order low-pass filter having a time constant of 300 µs and amplified 10 k-fold by a lock-in amplifier (MFLI 500 kHz Lock-in Amplifier, Zurich Instruments AG). The signal was subsequently digitized using a data acquisition board (NI USB-6351, National Instruments Corp., Austin, TX, USA) with a sample rate of 10 kS s^−1^ and a resolution of 16 bit that was controlled with a custom-written PC program (LabVIEW 2018, National Instruments Corp., Austin, TX, USA). This PC program facilitated the recording of the data streams and incorporated an optical correlation from a complementary metal-oxide-semiconductor (CMOS) camera with 5 MP resolution (GS3-U3-51S5M-C, Teledyne FLIR LLC, Wilsonville, OR, USA) mounted on a customized microscope (DM 2500 M, Leica Microsystems GmbH, Wetzlar, Germany) with 20× magnification.

The recorded data streams were analyzed with a custom state-event machine algorithm. After filtering the signal with a low-pass filter, the peaks of the characteristic four-peak signal were detected. More details can be found elsewhere [17].

### 3.2. Immunomagnetic Cell Labeling and Magnetic Flow Cytometry Measurements

Fresh venous blood was collected from healthy donors and stabilized with ethylenediaminetetraacetic acid (EDTA) (S-Monovette EDTA K3E 9 mL, Sarstedt AG & Co. KG, Nümbrecht, Germany). For immunomagnetic labeling of CD4^+^ T lymphocytes, a commercial labeling kit was used consisting of dextran-coated MNPs and anti-CD4 antibodies (EasySep Human CD4 Positive Selection Kit II, STEMCELL Technologies Canada Inc., Vancouver, BC, Canada). WB was incubated with a 9:1 ratio of the anti-CD4 antibody and MNPs each, e.g., 40 µL + 5 µL + 5 µL, for 30 min, unless stated differently, in a rotation shaker to avoid cell sedimentation.

For spiking immunomagnetically labelled CD4^+^ T lymphocytes into WB, the labelled sample was placed for 60 min in a magnetic separator, and subsequently the supernatant was removed carefully with a pipette. Finally, the extracted cells were resuspended in WB at different concentrations and diluted with Dulbecco’s Phosphate Buffered Saline (DPBS) (Sigma-Aldrich Corp., St. Louis, MO, USA) at an initial WB ratio of 1:10 or 1:50. The opaque sample was then measured with a flow rate of 30 µL min^−1^.

To simulate lower CD4^+^ T lymphocyte concentrations, WB was diluted with native plasma prior to mixing with the labeling reagents. Just before the measurement, the sample was diluted at a ratio of 1:30 with DPBS and measured for 3 min with a flow rate of 25 µL min^−1^.

### 3.3. Optical Reference Measurements

Reference concentrations of the CD4^+^ T lymphocytes in the WB samples were determined with an optical flow cytometer (MACSQuant Analyzer 10, Miltenyi Biotec B.V. & Co. KG, Bergisch Gladbach, Germany). Therefore, 20 µL WB was stained with 1 µL each of anti-CD4 antibody conjugated with FITC fluorophore (REAfinity, Miltenyi Biotec B.V. & Co. KG), anti-CD3 antibody conjugated with APC-Vio 770 fluorophore (REAfinity, Miltenyi Biotec B.V. & Co. KG), and anti-CD45 antibody conjugated with VioBlue fluorophore (REAfinity, Miltenyi Biotec B.V. & Co. KG). After 30 min incubation, the sample was diluted at a ratio of 1:30 with MACSQuant Running Buffer (Miltenyi Biotec B.V. & Co. KG) and immediately measured.

## 4. Results and Discussion

### 4.1. Cellular Background Impact on Determined Cell Concentrations with MFC

**Figure 2:**
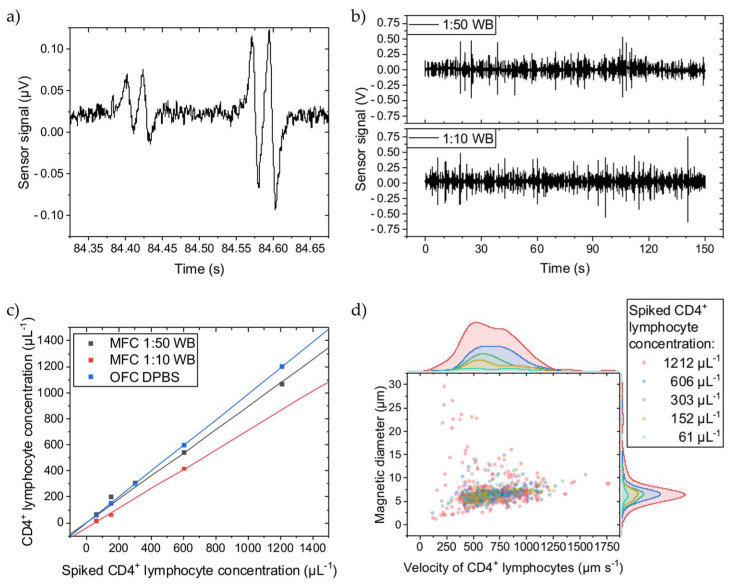
CD4^+^ T lymphocytes were spiked into WB diluted to 1:50 or 1:10 with DPBS, quantified with MFC, and compared to OFC. (**a**) Two typical four-peak sensor signals from immunomagnetically labelled CD4^+^ T lymphocytes showing different amplitudes. (**b**) Sensor signal stream for two WB dilutions. (**c**) The concentration measurements show a linear dependence for both dilutions. The slope of the linear fits for the 1:50 and 1:10 dilutions are 0.10 and 0.24 less than for the OFC reference fit. All fits have an adjusted R^2^ ≥ 0.99. (**d**) For the 1:50 WB dilution, the cell rolling velocity over the Wheatstone half-bridge at a flow rate of 30 µL min^−1^ was evaluated, and their magnetic diameter was inferred. Histograms of the scattered data are plotted on the right and top axes, respectively. The cell velocity is similarly distributed independent of the spiked CD4^+^ T lymphocyte concentration except for the highest concentration, where cells with higher and lower velocities than for the other concentrations can be found. The overall mean cell velocity is 687 µm s^−1^ with a CV of 0.34. Similarly, larger and smaller cell magnetic diameters were determined only for the 1212 µL^−1^ CD4^+^ T lymphocyte concentration. The overall mean cell magnetic diameter is 6.9 µm with a CV of 0.41.

The immunomagnetically labelled CD4^+^ T lymphocyte generates a distinct four-peak signal when passing over the Wheatstone half-bridge (Figure 2a). The time between two matching peaks correlates with the cell’s velocity passing the sensors. The signal amplitudes correlate with the MNP load on the cell, showing different cell labeling qualities. The signal-to-noise ratio of the first signal of 2.1 is just sufficiently high to ensure a robust signal detection analysis. In Figure 2b, representative signal streams can be compared for the 1:10 and 1:50 WB dilutions with spiked-in labelled CD4^+^ T lymphocytes showing no significant differences in signal frequency or amplitudes. The different cellular backgrounds dominated by the erythrocytes do not impair the four-peak signal.

However, when spiking different concentrations of labelled CD4^+^ T lymphocytes into different WB dilutions, the determined concentrations with MFC vary (Figure 2c). With more cellular background, the concentration decreases, meaning that not all immunomagnetically labelled cells pass over the GMR sensors. Since the sensors are on the bottom of the channel, the labelled cells must travel maximally the complete distance of the channel height (150 µm) before reaching the sensors. Thus, a higher cellular background creates more hindrances for the labelled CD4^+^ T lymphocytes, counteracting the magnetic force. While longitudinally transported through the channel, the labelled CD4^+^ T lymphocytes bump into other cells, primarily erythrocytes, on their way towards the channel bottom and possibly never get close enough before passing the GMR sensors and, therefore, do not get detected. To give a reference, in 1:10 and 1:50 diluted WB are 4–6 × 10^5^ µL^−1^ and 8–12 × 10^4^ µL^−1^ of erythrocytes, respectively, and thus are several magnitudes higher in concentration than the spiked-in CD4^+^ T lymphocytes [28]. Compared to the reference determined with OFC, where the CD4^+^ T lymphocytes were suspended into DPBS, the MFC detects fewer cells. For the 1:50 dilution, only 10% less CD4^+^ T lymphocytes were counted, while the 1:10 dilution shows a loss of 24% compared to the OFC measurements. Assuming an undiluted WB hematocrit of 50%, the 1:10 and 1:50 dilution results in a hematocrit of 5% and 1%, respectively. The difference in dilution (from 1:50 to 1:10) is equivalent to an increase in hematocrit by 400%. While the hematocrit can vary between 37–52% in humans, it can change by 40% (from 37% to 52%) [29]. If scaling the increase in hematocrit from the dilution by factor 10 to resemble the human hematocrit change, and also assuming linear scaling for the hematocrit-induced CD4^+^ T lymphocyte loss (14%), a 1.4% error in CD4^+^ T lymphocyte concentration with MFC from the patient’s hematocrit variance can be estimated.

Figure 2d depicts the CD4^+^ T lymphocyte velocity when passing over the Wheatstone half-bridge and their magnetic diameters. The magnetic diameter can be directly inferred from the temporal distance between a GMR sensor’s minimum and maximum signal peak distance by multiplication with the cell velocity [17]. Independent of the spiked CD4^+^ T lymphocyte concentration, they are similarly distributed except for the highest concentration of 1212 µL^−1^. In more detail, the amount of faster cells increases with higher concentrations. The number of cell interaction events at the tip of the magnetic rail probably increases so that bumping accelerates the cell. Additionally, minor signal analysis errors might contribute to this variance. The overall mean velocity is 687 µm s^−1^ with a coefficient of variance (CV) of 0.34 at a 30 µL min^−1^ flow rate, and the mean magnetic diameter is 6.9 µm with a CV of 0.41.

In brief, a higher cellular background reduces the retrieved CD4^+^ T lymphocyte concentration with MFC. However, over the 61–1212 µL^−1^ concentration range, MFC provides high linearity for quantifying immunomagnetically labelled CD4^+^ T lymphocytes irrespective of the cellular background and with negligible impact of patient’s hematocrit variance.

### 4.2. Simulating HIV Patient’s CD4^+^ T Lymphocyte Concentrations

**Figure 3:**
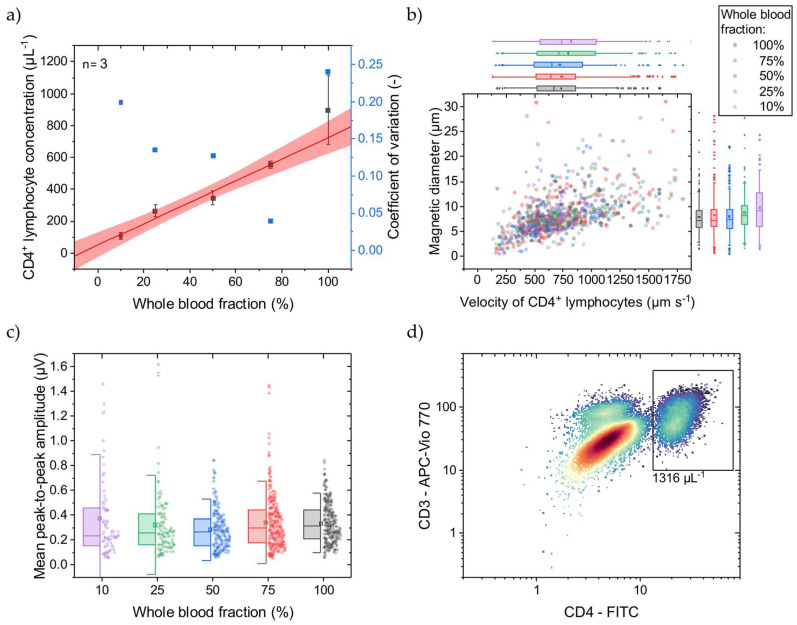
WB was diluted with native plasma to adjust the CD4^+^ T lymphocyte concentration before immunomagnetic labeling. (**a**) With a lower WB fraction, a lower concentration of CD4^+^ T lymphocytes is determined with the MFC. The linear fit (adjusted R^2^ = 0.98) and 95% prediction band are displayed in red. Only for undiluted WB (100%) does the CD4^+^ T lymphocyte concentration show high variance compared to the lower WB fractions. (**b**) For the different WB fractions, the cell rolling velocity over the Wheatstone half-bridge at a flow rate of 25 µL min^−1^ was evaluated, and their magnetic diameter was inferred. Box plots of the scattered data with an interquartile range of 50% and whiskers with lengths of 1.5× standard deviations (SD) are plotted on the right and top axes, respectively. For lower WB fractions, the mean cell velocity increases from 731 µm s^−1^ with an SD of 330 µm s^−1^ at 100% WB fraction to 822 µm s^−1^ with an SD of 443 µm s^−1^ at 10% WB fraction. Similarly, the mean cell magnetic diameter increases from 7.9 µm with an SD of 3.3 µm at 100% WB fraction to 9.8 µm with an SD of 5.0 µm at 10% WB fraction. (**c**) The mean peak-to-peak signal amplitudes with respective SDs, representing the MNP load per CD4^+^ T lymphocyte, increase with smaller WB fractions. The box plots show an interquartile range of 50% and whiskers with lengths of 1.5× SDs. (**d**) An exemplary reference OFC concentration of CD4^+^ T lymphocytes at 100% WB fraction indicated by the black rectangle.

Since we only had access to healthy donor blood samples, we simulated decreased CD4^+^ T lymphocyte concentrations similar to in vivo by diluting the WB samples prior to immunomagnetic labeling with their own, native plasma, respectively. This resulted in 10–100% WB fractions with corresponding CD4^+^ T lymphocyte concentrations. At 100% WB fraction, the CD4^+^ T lymphocyte concentration was determined with OFC, as exemplarily shown in Figure 3d, to be on average 1388 µL^−1^ with an SD of 74 µL^−1^ (*n* = 3). In Figure 3a, the CD4^+^ T lymphocyte concentrations determined with MFC are displayed, covering a mean range of 109–898 µL^−1^ being relevant for HIV-treatment-related decisions [4]. As reflected by the fit, good linearity (adjusted R^2^ = 0.98) can be demonstrated with a narrow 95% prediction band. The CV decreases from 0.20 to 0.04 at 10% and 75% blood fractions, respectively. Towards lower concentrations, each cell event gets more weight on the final result, if the total measurement time is constant. Here, all measurements took 3 min, irrespective of the CD4^+^ T lymphocyte concentration. Normalizing the measurements to the amount of detected CD4^+^ T lymphocytes would probably minimize the bias. However, at undiluted WB (100%), the CV increases to 0.24, indicating a high variance. Since the immunomagnetic labeling takes place after adjusting the WB fraction, and hematocrit changes show a minor impact on the CD4^+^ T lymphocyte concentration measurement, it is assumed that the quantitative immunomagnetic cell labeling can vary highly under this condition. In contrast, labeling with fluorophores and quantification with OFC gives a CV of approx. 0.05 at undiluted WB (100%).

Comparing MFC to OFC, the determined concentration at 100% WB fraction is 29% less with MFC than with OFC. While the MFC shows good linearity, a calibration line can compensate for this offset. Adding magnetic reference beads in the size of cells and known concentration to the sample before the measurements start could be an internal standard. Different magnetic moments and diameters can facilitate discrimination between immunomagnetically labeled CD4^+^ T lymphocytes and reference beads.

Towards lower WB fractions, the CD4^+^ T lymphocyte velocity, when passing the Wheatstone half-bridge, and magnetic diameter increase (Figure 3b). This increase can be attributed to CD4^+^ T lymphocyte aggregates forming over the magnetic rails. These cell aggregates protrude further into the parabolic flow profile and thus pass the sensors at a higher velocity. Investigating the signal amplitudes, they also increase with lower WB fraction (Figure 3c). These higher signal amplitudes could come from a higher MNP load per cell favored by better diffusion of the MNPs at lower cellular backgrounds and fewer CD4^+^ T lymphocytes. Additionally, the excess of MNPs increases for lower CD4^+^ T lymphocyte concentrations, promoting the formation of MNP aggregates that create high signal amplitudes when passing over the GMR sensors.

In summary, CD4^+^ T lymphocyte concentrations can be determined with MFC in the relevant range of ~100 µL^−1^ to >800 µL^−1^ with sufficient linearity. However, labeling reproducibility in undiluted WB needs to be optimized, especially regarding HIV patients, where the hematocrit is at physiological conditions during immunomagnetic labeling.

## 5. Conclusions

Immune status assessments by quantification of CD4^+^ T lymphocytes play an essential role in HIV treatment with a clinical need for POCT accessibility. In this work, we presented a fully integrated POCT workflow from sample acquisition over immunomagnetic cell labeling to non-optical probing of single cells until automated data analysis. To demonstrate its feasibility, we investigated quantitative MFC under WB conditions to determine CD4^+^ T lymphocyte concentrations in the clinically relevant range of approx. 60–1200 µL^−1^. With spiked immunomagnetically labelled CD4^+^ T lymphocytes at different concentrations into WB of different dilutions, we showed good linearity regarding quantitative concentration measurements with MFC. By simulating HIV patients covering two log scales of CD4^+^ T lymphocyte concentrations by diluting WB with their respective native plasma prior to immunomagnetic labeling, we demonstrate that labeling in WB can work as well as subsequent CD4^+^ T lymphocyte quantification. However, more understanding is needed for immunomagnetic labeling with MNPs in undiluted whole blood. By integrating this work into a single-use cartridge, POCT needs would be met, potentially allowing for a more cost-efficient and more easily accessible HIV patient treatment [30]. The World Health Organization’s target product profile for a CD4 POCT lists detailed test and system requirements, including its intended use, required performance, and operational characteristics [30]. Although we are aware of compulsory in-depth validation and performance testing of our CD4 POCT for regulatory approval, we already match numerous requirements from a technological and workflow perspective, e.g., sample preparation, sample volume and type, CD4 result, and operator skills. While we are further improving the workflow integration, preliminary results with a self-built cartridge and small footprint reader were already promising.

## Data Availability

The raw data supporting the conclusions of this article will be made available by the authors on request.

## Supplementary Materials

The following supporting information can be downloaded at: https://www.mdpi.com/article/10.3390/mi15040520/s1. Figure S1. Laboratory-scale magnetic flow cytometry setup. On top of the non-magnetic pole is the permanent magnet fixed that can be precisely positioned relative to the GMR sensors with the *x*/*y*/*z*-stage. The sensor chip (assembled PCB, Si chip, and PDMS fluidic) is mounted on a PCB holder. The Helmholtz coils are used for hysteresis measurements to optimize the magnet’s position for maximum GMR sensor sensitivity. Once an optimal magnet position is found, no further hysteresis measurements are needed as long as the relative position of the magnet and GMR sensors are not changed. The syringe pump provides a pulsation-free sample flow [31]. The data acquisition board digitizes the signal from the lock-in amplifier and forwards it to a computer. The microscope and camera are used for control purposes only. Figure S2. Details of the magnetic flow cytometer setup. (a) The non-magnetic PCB holder keeps the sensor chip centrally in the Helmholtz coils. For optimizing the permanent magnet’s position, the Helmholtz coils generate an alternating magnetic flux density between ±15 mT, resulting in hysteresis curves measured using the GMR sensor [17]. The scale bar represents 2 cm. (b) The permanent magnet is fixed on a non-magnetic pole. (c) The mounted sensor chip compromises PCB, Si chip, and PDMS fluidic. (b,c) The scale bars represent 1 cm. Figure S3. Sensor chip of the magnetic flow cytometer. (a) The PDMS fluidic compromises a straight channel of 150 µm height and 700 µm width. The electrical connection of the GMR sensors is facilitated by wire bonding between PCB and Si chip bond pads. The scale bar represents 5 mm. (b) The white arrow indicates the sample flow direction. Upon magnetic attraction of magnetized cells, magnetic rails focus these cells on the GMR sensors [18]. The GMR sensor array consists of multiple GMR sensors in a Wheatstone half-bridge configuration with different GMR sensor distances [17]. The scale bar represents 500 µm.
