# Supplementary material for: Towards a Point-of-Care Test of CD4+ T Lymphocyte Concentrations for Immune Status Monitoring with Magnetic Flow Cytometry
Source: Micromachines (Basel). 2024 Apr 13;15(4):520. doi: 10.3390/mi15040520 (PMC11051966; doi:10.3390/mi15040520)
Supplement: Supplementary file 1 [file micromachines-15-00520-s001.zip › micromachines-2918812-supplementary.pdf]

micromachines

Supplementary Materials

Towards a Point-of-Care Test of CD4⁺ T Lymphocyte Concentrations for Immune Status Monitoring with Magnetic Flow Cytometry

Moritz Leuthner [1,*], Mathias Reisbeck [1], Michael Helou [2] and Oliver Hayden [1,*]

[1] Heinz-Nixdorf-Chair of Biomedical Electronics, School of Computation, Information and Technology & Munich Institute of Biomedical Engineering, Technical University of Munich, TranslaTUM, Einsteinstraße 25, 81675 Munich, Germany
[2] EarlyBio GmbH, Bottroper Weg 2, 13507 Berlin, Germany
* Correspondence: moritz.leuthner@tum.de (M.L.); oliver.hayden@tum.de (O.H.)

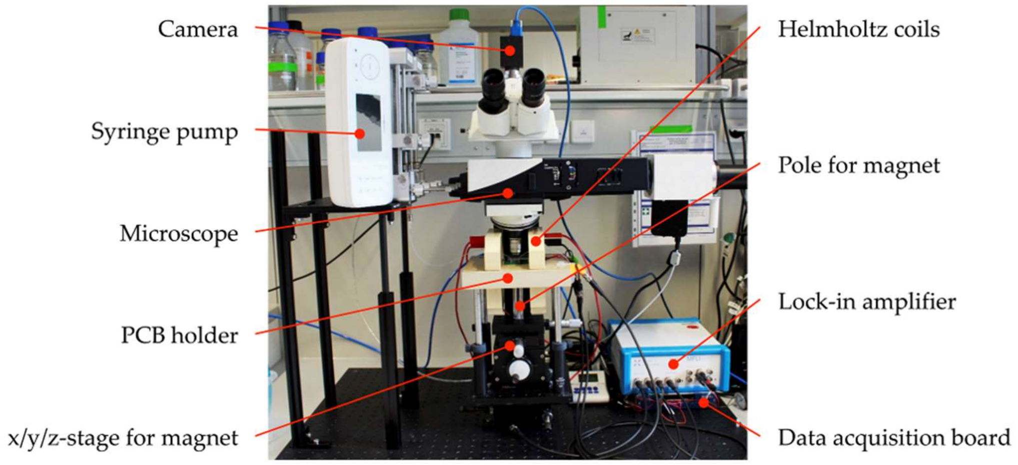

Figure S1. Laboratory-scale magnetic flow cytometry setup. On top of the non-magnetic pole is the permanent magnet fixed (not shown) that can be precisely positioned relative to the GMR sensors with the *x*/*y*/*z*-stage. The sensor chip (assembled PCB, Si chip, and PDMS fluidic) is mounted on a PCB holder. The Helmholtz coils are used for hysteresis measurements to optimize the magnet's position for maximum GMR sensor sensitivity. Once an optimal magnet position is found, no further hysteresis measurements are needed as long as the relative position of the magnet and GMR sensors are not changed. The syringe pump provides a pulsation-free sample flow [31]. The data acquisition board digitizes the signal from the lock-in amplifier and forwards it to a computer. The microscope and camera are used for control purposes only.

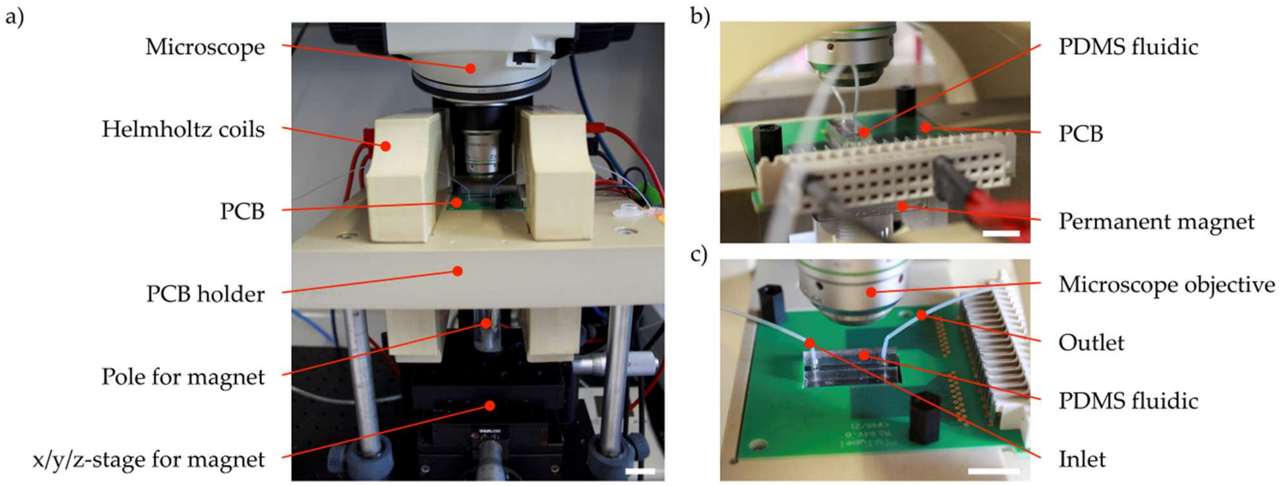

Figure S2. Details of the magnetic flow cytometer setup. (**a**) The non-magnetic PCB holder keeps the sensor chip centrally in the Helmholtz coils. For optimizing the permanent magnet's position, the Helmholtz coils generate an alternating magnetic flux density between ±15 mT, resulting in hysteresis curves measured using the GMR sensor [17]. The scale bar represents 2 cm. (**b**) The permanent magnet is fixed on a non-magnetic pole. (**c**) The mounted sensor chip compromises PCB, Si chip, and PDMS fluidic. (**b**,**c**) The scale bars represent 1 cm.

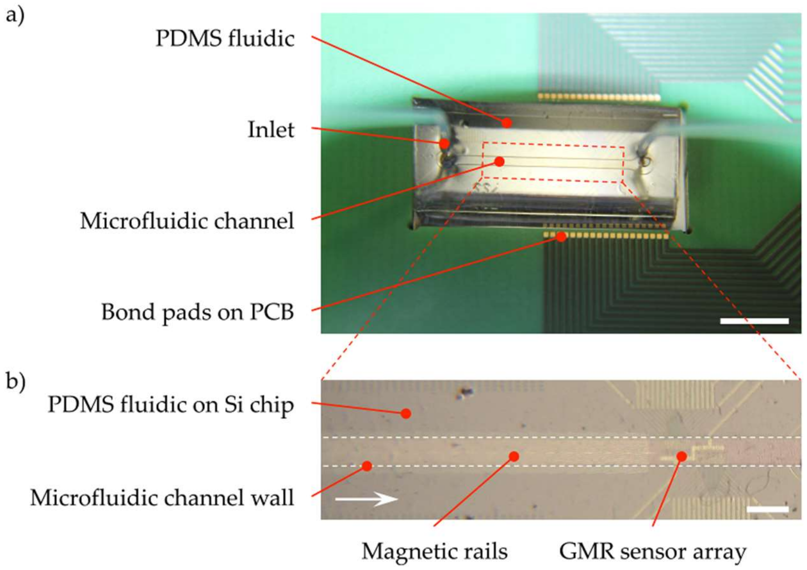

Figure S3. Sensor chip of the magnetic flow cytometer. (**a**) The PDMS fluidic compromises a straight channel of 150 µm height and 700 µm width. The electrical connection of the GMR sensors is facilitated by wire bonding between PCB and Si chip bond pads. The scale bar represents 5 mm. (**b**) The white arrow indicates the sample flow direction. Upon magnetic attraction of magnetized cells, magnetic rails focus these cells on the GMR sensors [18]. The GMR sensor array consists of multiple GMR sensors in a Wheatstone half-bridge configuration with different GMR sensor distances [17]. The scale bar represents 500 µm.

References

17. Reisbeck, M.; Helou, M.J.; Richter, L.; Kappes, B.; Friedrich, O.; Hayden, O. Magnetic fingerprints of rolling cells for quantitative flow cytometry in whole blood. *Sci. Rep.* **2016**, *6*, 32838.
18. Helou, M.J.; Reisbeck, M.; Tedde, S.F.; Richter, L.; Bär, L.; Bosch, J.J.; Stauber, R.H.; Quandt, E.; Hayden, O. Time-of-flight magnetic flow cytometry in whole blood with integrated sample preparation. *Lab Chip* **2013**, *13*, 1035.
31. Leuthner, M.; Hayden, O. Grease the gears: How lubrication of syringe pumps impacts microfluidic flow precision. *Lab Chip* **2024**, *24*, 56–62.
